# Novel Intramedullary-Fixation Technique for Long Bone Fragility Fractures Using Bioresorbable Materials

**DOI:** 10.1371/journal.pone.0104603

**Published:** 2014-08-11

**Authors:** Takanobu Nishizuka, Toshikazu Kurahashi, Tatsuya Hara, Hitoshi Hirata, Toshihiro Kasuga

**Affiliations:** 1 Department of Hand Surgery, Nagoya University Graduate School of Medicine, Nagoya, Japan; 2 Department of Frontier Materials, Nagoya Institute of Technology, Nagoya, Japan; University of Akron, United States of America

## Abstract

Almost all of the currently available fracture fixation devices for metaphyseal fragility fractures are made of hard metals, which carry a high risk of implant-related complications such as implant cutout in severely osteoporotic patients. We developed a novel fracture fixation technique (intramedullary-fixation with biodegradable materials; IM-BM) for severely weakened long bones using three different non-metallic biomaterials, a poly(l-lactide) (PLLA) woven tube, a nonwoven polyhydroxyalkanoates (PHA) fiber mat, and an injectable calcium phosphate cement (CPC). The purpose of this work was to evaluate the feasibility of IM-BM with mechanical testing as well as with an animal experiment. To perform mechanical testing, we fixed two longitudinal acrylic pipes with four different methods, and used them for a three-point bending test (N = 5). The three-point bending test revealed that the average fracture energy for the IM-BM group (PLLA + CPC + PHA) was 3 times greater than that of PLLA + CPC group, and 60 to 200 times greater than that of CPC + PHA group and CPC group. Using an osteoporotic rabbit distal femur incomplete fracture model, sixteen rabbits were randomly allocated into four experimental groups (IM-BM group, PLLA + CPC group, CPC group, Kirschner wire (K-wire) group). No rabbit in the IM-BM group suffered fracture displacement even under full weight bearing. In contrast, two rabbits in the PLLA + CPC group, three rabbits in the CPC group, and three rabbits in the K-wire group suffered fracture displacement within the first postoperative week. The present work demonstrated that IM-BM was strong enough to reinforce and stabilize incomplete fractures with both mechanical testing and an animal experiment even in the distal thigh, where bone is exposed to the highest bending and torsional stresses in the body. IM-BM can be one treatment option for those with severe osteoporosis.

## Introduction

Metallic implants such as locked plating or intramedullary nailing can instantaneously strengthen weakened long bone metaphyses; however, they sometimes cause complications such as cutout because the strength of the bone is much less than that of the metallic implants in severely osteoporotic patients [1]. We therefore developed a new technique, intramedullary-fixation with biodegradable materials (IM-BM), to instantaneously strengthen severely weakened long bone metaphyses, imitating vertebroplasty. Vertebroplasty [2] can immediately make the collapsed vertebrae stronger using calcium phosphate cement (CPC) [3]; however, there is no such procedure with CPC for long bone metaphyses due to the limited torsional and bending strength associated with CPC [3], [4]. We therefore combined a poly(l-lactide) (PLLA) woven tube with CPC based on a concept of reinforced concrete in order to strengthen severely weakened long bone.

Reinforced concrete is one of the most widely used modern building materials to strengthen the framework. Typical concrete has high resistance to compressive stresses (about 28 MPa); however, any appreciable tension (*e.g.,* due to bending) will break the microscopic rigid lattice, resulting in cracking and concrete separation. Reinforced concrete is a composite material made by inserting steel bars in concrete, and resists not only compression but also bending and torsional stresses.

We also combined a nonwoven polyhydroxyalkanoates (PHA) fiber mat to prevent cement leakage from the fracture site. PHA are biodegradable materials and show excellent extendibility.

The purpose of this work was to evaluate the feasibility of IM-BM in both mechanical testing and animal experiment models. The present work demonstrated that IM-BM was strong enough to reinforce and stabilize incomplete fractures with both mechanical testing and an animal experiment even in the distal thigh, where bone is exposed to the highest bending and torsional stresses in the body.

## Materials and Methods

### Ethics statement

The Institutional Committee for Animal Care of Nagoya University approved the experimental protocol (reference number: 25166).

### Intramedullary-fixation with Biodegradable Materials (IM-BM)

**Figure 1:**
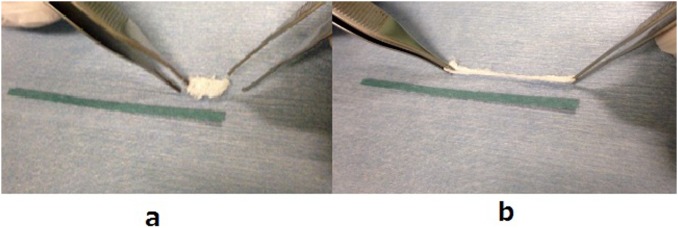
Expandability of PHA fiber mat. Before elongation (a) and after elongation (b).

**Figure 2:**
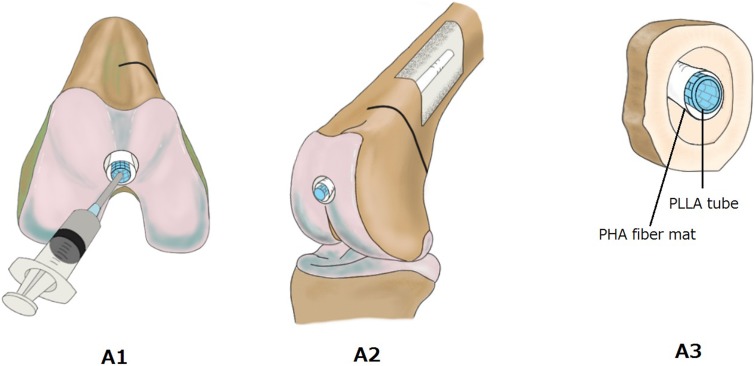
IM-BM procedures. View of the distal femur (A1, A2) and schema of bone cross section (A3) before CPC injection. PLLA: PLLA woven tube; PHA: PHA fiber mat; CPC: calcium phosphate cement.

**Figure 3:**
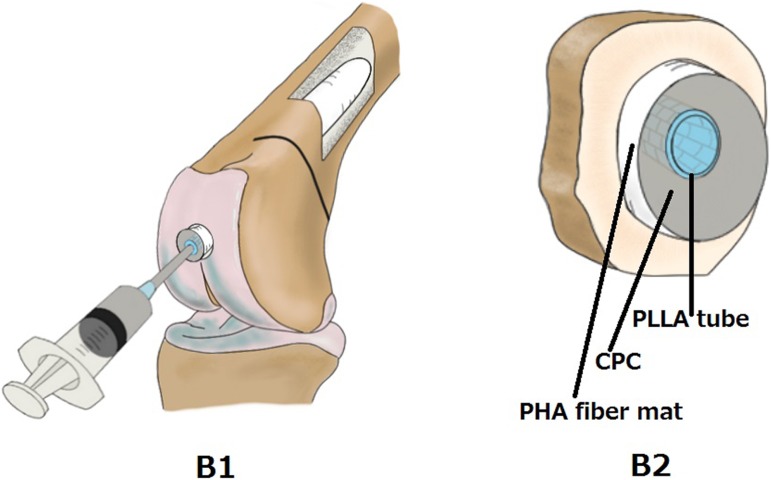
IM-BM procedures. View of the distal femur (B1) and bone cross section schema (B2) after CPC injection. PLLA: PLLA woven tube; PHA: PHA fiber mat; CPC: calcium phosphate cement.

The procedure for IM-BM begins with reaming the intramedullary cavity and inserting a PLLA woven tube into the cavity, followed by injection of CPC paste both inside and outside the tube using a syringe. However, massive CPC paste leakage occurs from the fracture site, and it appears likely to inhibit bone union. Therefore, we use a nonwoven PHA fiber mat to prevent CPC leakage. The 3-hydroxybutyrate (3HB) and 4-hydroxybutyrate (4HB) copolymers (poly [P](3HB-co-4HB)) used in the PHA fiber mat in this work demonstrate both strength and flexibility (Figure 1). The nonwoven PHA fiber mat can prevent CPC leakage. Therefore, when we injected the CPC paste inside and outside the PLLA woven tube, the nonwoven PHA fiber mat was expanded until it fit the cavity (Figures 2 and 3).

### Preparation of PLLA Woven Tube

Nipro Co., Ltd. (Tokyo, Japan) produced the PLLA woven tube used in this study. First, a plain-stitch fabric was knitted using PLLA monofilaments (0.2 mm in diameter). Then, three sheets of fabric were stacked, and formed into a cylindrical shape by painting with dichloromethane (Wako Junyaku Kogyo Co., Ltd., Osaka, Japan), with a resulting external and internal diameter of 10 and 7 mm, respectively. Finally, they were cut and shortened to 65 mm in length for mechanical testing. Smaller size PLLA woven tubes for the animal experiment, with external and internal diameters of 5 and 4 mm, respectively, were also prepared. They were cut and shortened to 38 mm in length. Our previous experiment indicated that the mean modulus of elasticity in bending for these tubes was 46.7 MPa.

### Preparation of a Nonwoven PHA Fiber Mat

PHA represent a complex class of biopolymers consisting of various hydroxyalkanoic acids. Microorganisms synthesize PHA as storage compounds for energy [5]. PHA exhibit biodegradable, biocompatible, thermoplastic and elastomeric properties once extracted from the cells [6]. Attempts have been made for many years to develop PHA applications in medical devices [7]. Polymerization of 4HB with other hydroxyl acids such as 3HB can produce elastomeric compositions at moderate 4HB contents (15%–35%), and relatively hard rigid polyesters at lower 4HB contents [7]. 3HB and 4HB copolymer (P(3HB-co-4HB)) at 18% 4HB content (G5 JAPAN Co., Ltd., Osaka, Japan) was used in our work as a tool to prevent CPC leakage.

**Figure 4:**
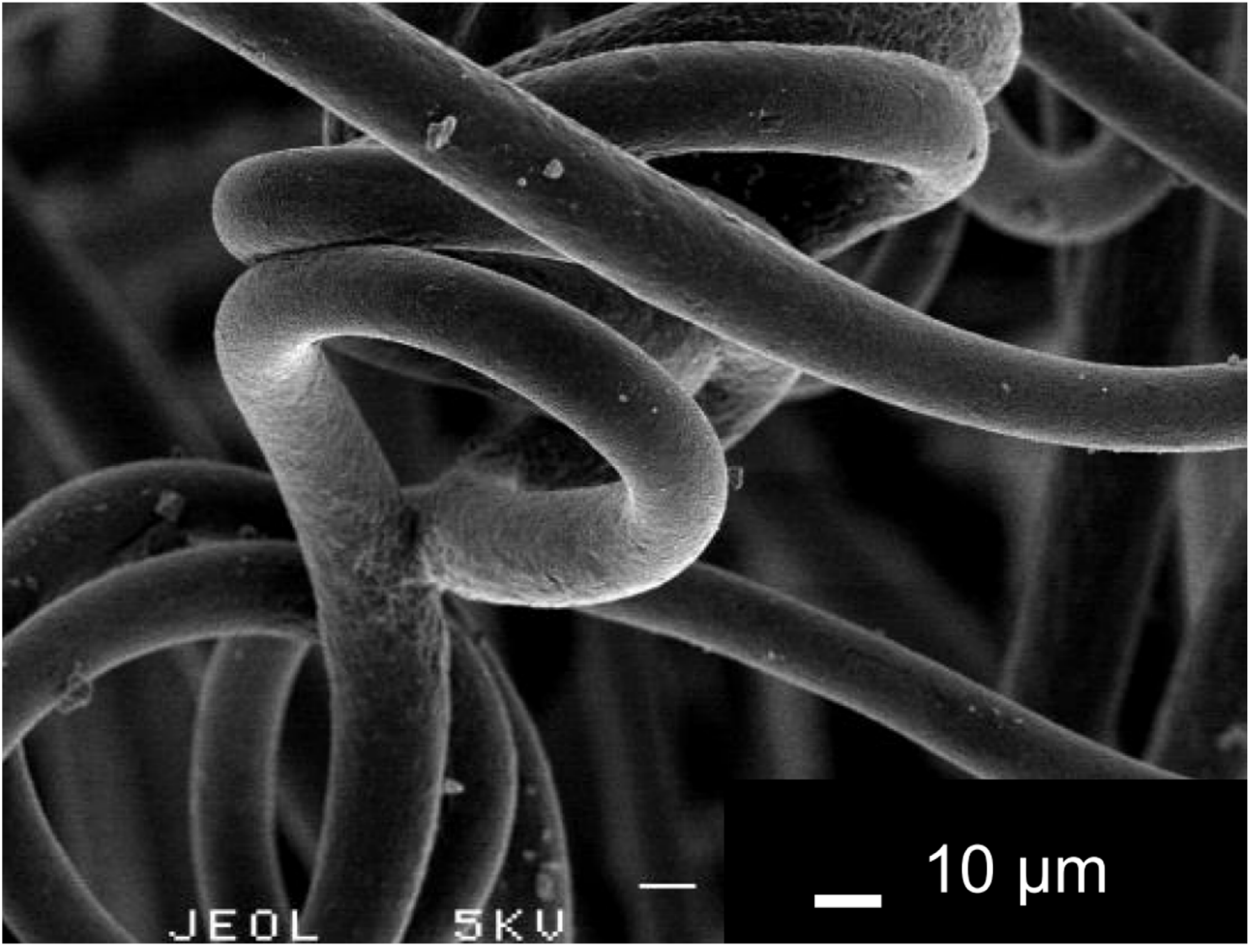
Scanning Electron Microscopy (SEM) photograph of a nonwoven PHA fiber mat. A nonwoven PHA fiber mat, consisting of microfibers with diameters of approximately 10 µm.

Electrospinning is a process that can generate a polymer fiber mat with high flexibility and porosity [8], [9]. A porous material with continuous pore structure is expected to be useful for implants used in the regeneration of damaged tissue, because the pore would allow penetration of nutriments and/or ingrowth of tissues, blood vessels, and cells [10]. The nonwoven fiber mat consisting of a biodegradable polymer may be one of the best biomaterial candidates, because the interlocking fibers easily form large connective pores [11]. A nonwoven PHA fiber mat with 3HB and 4HB copolymers was prepared using an electrospinning method. First, 2 g of PHA powder consisting of P(3HB-co-4HB) copolymers containing 18% 4HB (G5 JAPAN Co.) were dissolved in chloroform at 6 wt% to prepare the solution for electrospinning. The samples were then spun on the electrospinning unit (NEU, Kato Tech Co., Kyoto, Japan). The solution for electrospinning was loaded into a glass syringe and pushed out at a flow rate of 30 µl/min through a metallic needle (22 gauge) that was connected to a +10 kV electrical field at room temperature and approximately 55% relative humidity. The fibers were collected on an aluminum drum rotating at 2000 mm/min, traversing at 100 mm/min, and positioned 80 mm from the tip of the needle. The nonwoven PHA fiber mat, consisting of microfibers with diameters of approximately 10 µm (Figure 4), was produced with a final thickness of 100 µm after spinning for 120 min. The resulting nonwoven PHA fiber mat showed excellent expandability (Figure 1); it did not fracture easily even when the CPC paste was injected to expand it (Figure 3).

### CPC Preparation

The CPC used in the present work was Biopex-R^®^ (advanced type) (BPRad, HOYA Co., Ltd., Tokyo, Japan). The CPC powder consisted of α-tricalcium phosphate (α-Ca_3_(PO_4_)_2_; α-TCP), tetracalcium phosphate (Ca_4_(PO_4_)_2_O; TECP), dicalcium phosphate dihydrate (CaHPO_4_·2H_2_O), hydroxyapatite (Ca_10_(PO_4_)_6_(OH)_2_; HAp) and magnesium phosphate (Mg_3_(PO_4_)_2_). The malaxation liquid was composed of sodium chondroitin sulfate, disodium succinate ((CH_2_COONa)_2_), sodium hydrogensulfite (NaHSO_3_) and water (H_2_O). The CPC powder was mixed with the malaxation liquid, turning it into a paste, which hardened over time via hydration to form a hydroxyapatite structure [12]. Twelve grams of powder and 4 ml of malaxation liquid were used in the present mechanical testing and animal experiment. BPRad takes only 1 day to reach maximum compressive strength after kneading of the powder and malaxation liquid mixture.

### Mechanical Testing

**Figure 5:**
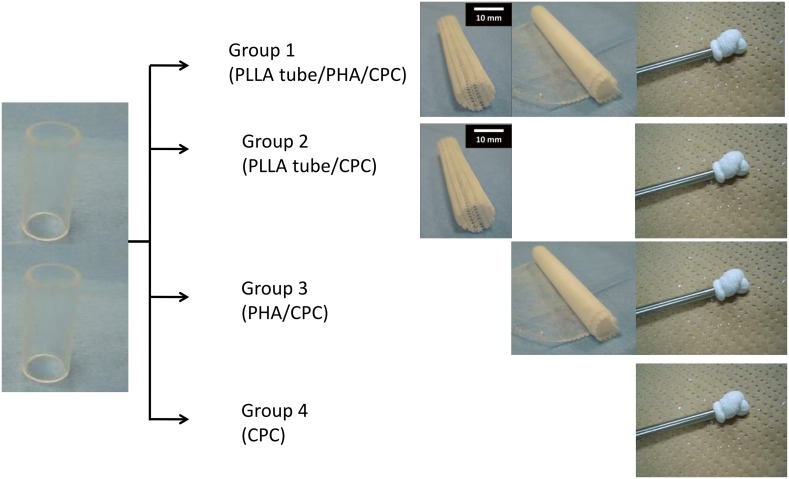
Method of mechanical testing. Two acrylic pipes placed longitudinally were fixed in four groups by the different method. Pictures of group 1 show a PLLA woven tube, a nonwoven PHA fiber mat, and CPC paste sequentially from the left.

Five profile specimens were tested for each sample composition. As shown in Figure 5, we fixed two longitudinal acrylic pipes with four different methods. For group 1, the PLLA woven tube was wrapped by the nonwoven PHA fiber mat, and it was inserted into two acrylic pipes placed longitudinally. The outer diameter of the pipe was 16 mm, and the length of each pipe was 35 mm. The powder and the liquid in the incubator (NTT-2200, EYELA, Tokyo, Japan) were warmed to 30°C before the injection. Just after kneading, the CPC paste was injected inside and outside the PLLA woven tube using a syringe. After the injection, the specimens were submerged in simulated body fluid (Na^+^ 142.0, K^+^ 5.0, Mg^2+^ 1.5, Ca^2+^ 2.5, Cl^−^ 148.8, HCO_3_^−^ 4.2, PO_4_^2−^ 1.0, and SO_4_^2−^ 0.5 mM and buffered at pH 7.40 with trishydroxymethyl-aminomethane) [13] maintained at 37°C. The CPC started to harden gradually. After 10 min, a three-point bending test was performed using 858 Mini Bionix II (MTS, Eden Prairie, MN) following the Japanese Industrial Standard (JIS) K 7074. The cross-head speed was 0.5 mm/min, and the support span was 60 mm. For group 2, the PLLA woven tube was inserted into two acrylic pipes placed longitudinally. Then, the CPC paste was injected inside and outside the PLLA woven tube. For group 3, the cylindrical shape of the nonwoven PHA fiber mat was inserted into acrylic pipes. Then, the CPC paste was injected into the nonwoven PHA fiber mat. Only the CPC paste was injected into acrylic pipes for group 4.

The maximum flexural strength was determined using the following equation:
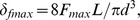
where *δ_fmax_* is the maximum flexural strength (MPa), *F_max_* is the maximum load (N), *L* is the support span (mm), and *d* is the diameter of the specimen (mm).

The modulus of elasticity in bending was determined using the following equation:
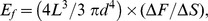
where *E_f_* is the modulus of elasticity in bending (MPa), *L* is the support span (mm), *d* is the diameter of the specimen (mm), *ΔF* is the variation of the load (N), and *ΔS* is the variation of the central deflection (mm).

Fracture energy (J/m^2^) was determined using integral calculus from the beginning to the yield point at the stress–strain curve. A mean value and standard deviation were calculated concerning the δ_fmax_, the modulus of elasticity in bending, and the fracture energy.

All statistical analyses were conducted using SPSS version 19.0 (SPSS, Tokyo, Japan). Data from multiple groups were compared using a one-way analysis of variance (ANOVA). We performed multiple comparisons of the three treatment groups using the Bonferroni test when significant differences were detected. Significance levels of all the tests were established at *p*<0.05.

### Experimental Animal Model of Osteoporosis and Surgical Procedure

Sixteen skeletally mature, 8-month-old (3.0–3.5 kg body weight), female New Zealand white rabbits were used. Experimental rabbit osteoporosis models were made by performing bilateral ovariectomy followed by intramuscular injection with methylprednisolone sodium succinate (Solu-Medrol; Pfizer, Tokyo, Japan) at a dosage of 1 mg/kg/day for four consecutive weeks, as previously described by Castaneda et al. [14] The sixteen rabbits were then randomly allocated into four experimental groups. The first was an IM-BM group (PLLA woven tube + CPC + nonwoven PHA fiber mat). The second was a PLLA + CPC group. The third was a CPC group. The fourth was a Kirschner wire (K-wire) group.

All the PLLA woven tubes and the nonwoven PHA fiber mat used in our animal experiments were sterilized with ethylene oxide gas (20% by weight; CO_2_ 80% by weight) with 50% H_2_O at 45°C for 5 h. The rabbits were anesthetized with an intramuscular injection of ketamine hydrochloride (75 mg/kg body weight) and xylazine (10 mg/kg body weight) 8 weeks after the ovariectomy. The surgical site was shaved and prepared with a solution of Betadine (povidone-iodine). A lateral parapatellar incision was made, and the patella was medially dislocated. A half-round fracture was produced 10 mm proximal to the distal end of the femur with an electrical cutter to reproduce an insufficiency fracture. In the K-wire group, two 1.5-mm K-wires were inserted intramedullary from the intercondylar area 5 mm proximal from the edge of the intercondylar notch until penetrating the proximal bone cortex. Finally, the fascia and the skin wounds were closed. In the IM-BM group, PLLA + CPC group, and the CPC group, a drill hole was produced in the intercondylar area 5 mm proximal from the edge of the intercondylar notch using 1.5-mm K-wire. Next, an intramedullary cavity was reamed from 3 mm to 7 mm by the drill reamer, and the cavity was curetted.

In the IM-BM group, the PLLA tube wrapped with nonwoven PHA fiber mat was inserted into the cavity (Figure 2-A1, A2, A3). Next, CPC paste was injected into the lumen of the PLLA tube just after kneading the powder and malaxation liquid, which was preheated to 30°C in the incubator (Figure 3-B1, B2). The paste began to harden about 10 min after kneading. In the PLLA + CPC group, CPC paste was injected after insertion of the PLLA woven tube. In the CPC group, only CPC paste was injected. A small amount of CPC paste leaked from the fracture site in the PLLA + CPC group and the CPC group. In the IM-BM group, the PLLA + CPC group, and the CPC group, the fascia and the skin wounds were closed within 15 min after kneading of the CPC paste, and the rabbits began to bear weight a few hours after the operation.

Postoperative lesions were evaluated with the use of soft X-ray (SOFTEX, Yokohama, Japan) at weeks 1, 4, 8, 12, and 20. Lateral radiographs were made with an exposure of 45 kV, 10 mA, and 15 s. The soft X-ray data at postoperative week 1 from multiple groups were compared using a one-way ANOVA. When significant differences were detected in ANOVA, we performed multiple comparisons of the four treatment groups using the Bonferroni test. A significance level for the test was established at *p*<0.05.

With pentobarbital overdose, two of the four rabbits in the IM-BM group were sacrificed at week 20, and the other two in the IM-BM group were sacrificed at week 52. A 15 mm segment of the metaphyseal portion of the femur was removed for histological examination. The specimens were fixed with 0.2% glutaraldehyde for 24 h and then in 10% phosphate-buffered formalin for 48 h. The specimens were subsequently dehydrated in ethanol and embedded in methyl methacrylate (MMA). The embedded blocks were trimmed with a cutter and ground with abrasive paper. Thereafter, the sections were further ground to a final thickness of approximately 10 µm. Finally, the specimens were stained with hematoxylin and eosin, and examined under the microscope.

## Results

### Mechanical Testing

**Figure 6:**
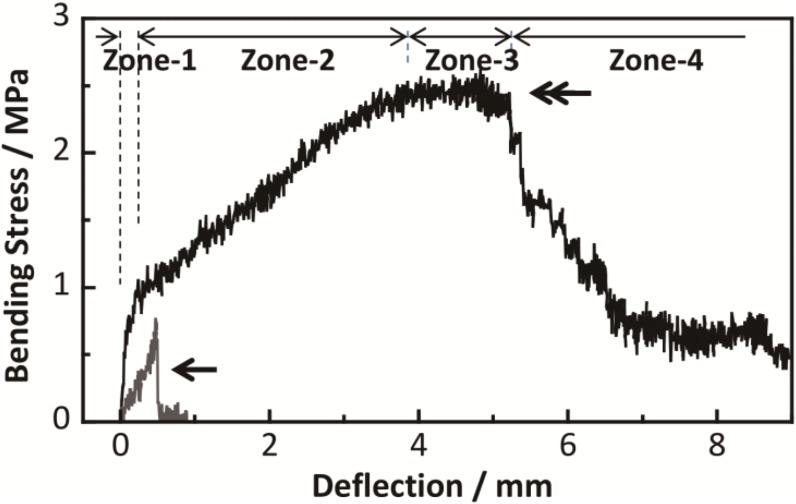
Result of mechanical testing. A representative stress–strain curve 10 min after the CPC injection in group 1 (double arrow) and group 4 (arrow).

Table 1 shows the mean maximum flexural strength values for each group at 10 min immediately following the CPC injection. The group 1 values (PLLA + CPC + PHA; 2.71±0.66 MPa) were significantly higher (*p*<0.001) than the values of group 3 (CPC + PHA; 0.79±0.23 MPa) and group 4 (CPC only; 0.52±0.24 MPa). Although there is no statistically significant difference, the values of group 1were higher than the values of group 2 (PLLA + CPC; 2.37±0.13 MPa) (*p* = 0.15). Table 1 also shows the average fracture energy at 10 min following the CPC injection. The group 1 values (PLLA + CPC + PHA; 1210±334 J/m^2^) were significantly higher (*p*<0.001) than the group 2 values (PLLA + CPC; 434±63 J/m^2^), the group 3 values (CPC + PHA; 19.4±6.4 J/m^2^) and the group 4 values (CPC only; 5.5±5.7 J/m^2^). Figure 6 shows the representative stress–strain curves for group 1 (PLLA + CPC + PHA) and group 4 (CPC only). In group 1, the curve can be categorized into four zones (zone 1: steep slope zone, zone 2: gentle slope zone, zone 3: almost flat slope zone, and zone 4: negative slope zone). In zones 1 and 2, the stress kept increasing and the curve did not drop until the end of zone 3. In contrast, in groups 3 and 4, the curve immediately reached the yield point. Once the curve reached the yield point, the curve dropped abruptly due to the fragmentation of the materials. The average moduli of elasticity in bending in group 1 were 179±89 MPa in zone 1 and 18.2±8.7 MPa in zone 2. The average moduli of elasticity in bending in groups 2, 3, and 4 were 144±31 MPa, 183±64 MPa and 223±69 MPa, respectively.

**Table 1:** The mean maximum flexural strength values and the mean fracture energy for each group.

	Group 1(PLLA tube/PHA/CPC)	Group 2(PLLA tube/CPC)	Group 3(PHA/CPC)	Group 4(CPC)
Maximum flexural strength(MPa)	*2.71±0.66	2.37±0.13	0.79±0.23	0.52±0.2
Fracture energy(J/m^2^)	*1210±334	434±63	19.4±6.4	5.5±5.7

*Significant difference (p<0.001) in the maximum flexural strength between group 1(*) and group 3/group 4 (Bonferroni test).

*Significant difference (p<0.001) in the fracture energy between group 1(*) and group 2/group 3/group 4 (Bonferroni test).

PLLA: poly(l-lactide); PHA: polyhydroxyalkanoates; CPC: calcium phosphate cement.

### Animal Experiments

**Figure 7:**
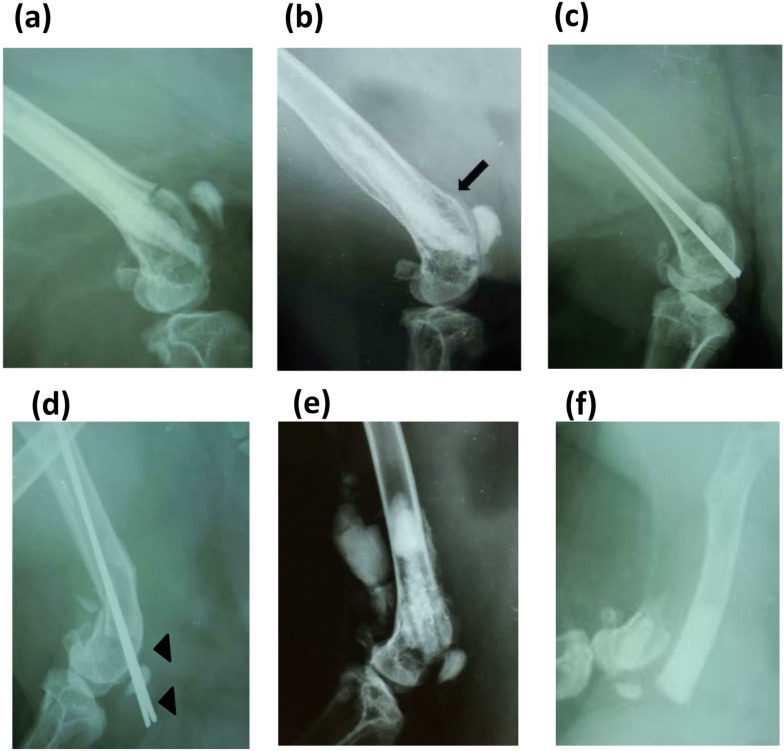
Representative postoperative radiographs. Representative postoperative radiographs in the IM-BM group (PLLA + CPC + PHA) at week 0 (a) and week 8 (b). (b) The fracture site obtained complete bony union (arrow). Representative postoperative radiographs in the Kirschner wire group at week 0 (c) and week 1 (d). (d) Cutout happened at the fracture site (arrowhead). Representative postoperative radiograph in the PLLA + CPC group at week 0 (e). (e) It showed CPC leakage from the fracture site. Representative postoperative radiograph in the CPC group at week 1 (f). (f) It showed fracture displacement.

Soft X-ray photographs revealed that there were no fracture displacements (0/4) over the entire postoperative period in the IM-BM group (Figure 7a), whereas two of four rabbits in PLLA + CPC group, three of four rabbits in the CPC group (Figure 7f), and three (including one cutout) of four rabbits in the K-wire group (Figure 7d) had fracture displacements at postoperative week 1. Although statistical analysis did not indicate a significant difference, IM-BM group had fewer fracture displacements than other groups (PLLA + CPC, CPC, K-wire) (*p* = 0.35, 0.143, 0.143, respectively: all Bonferroni multiple comparison test), as shown in Table 2. Figure 7b shows that all rabbits in the IM-BM group achieved bony union in 8 weeks. There were no postoperative infections or clinical signs of implant reaction in the IM-BM group. On the other hand, all rabbits in the PLLA + CPC group showed CPC leakage from the fracture site (Figure 7e), and three of four rabbits in the CPC group had wound dehiscence within the first postoperative week.

**Table 2:** Fracture displacement rate in four groups at postoperative week 1.

	IM-BM group	PLLA + CPC group	CPC group	K-wire group
Fracture displacement rate	0/4	2/4	3/4	3/4

IM-BM group had fewer fracture displacements than the PLLA + CPC, the CPC, and the K-wire groups although statistical analysis did not indicate a significant difference (*p* = 0.35, 0.143, 0.143, respectively: all Bonferroni multiple comparison test).

IM-BM: intramedullary-fixation with biodegradable materials; CPC: calcium phosphate cement; K-wire: Kirschner wire.

**Figure 8:**
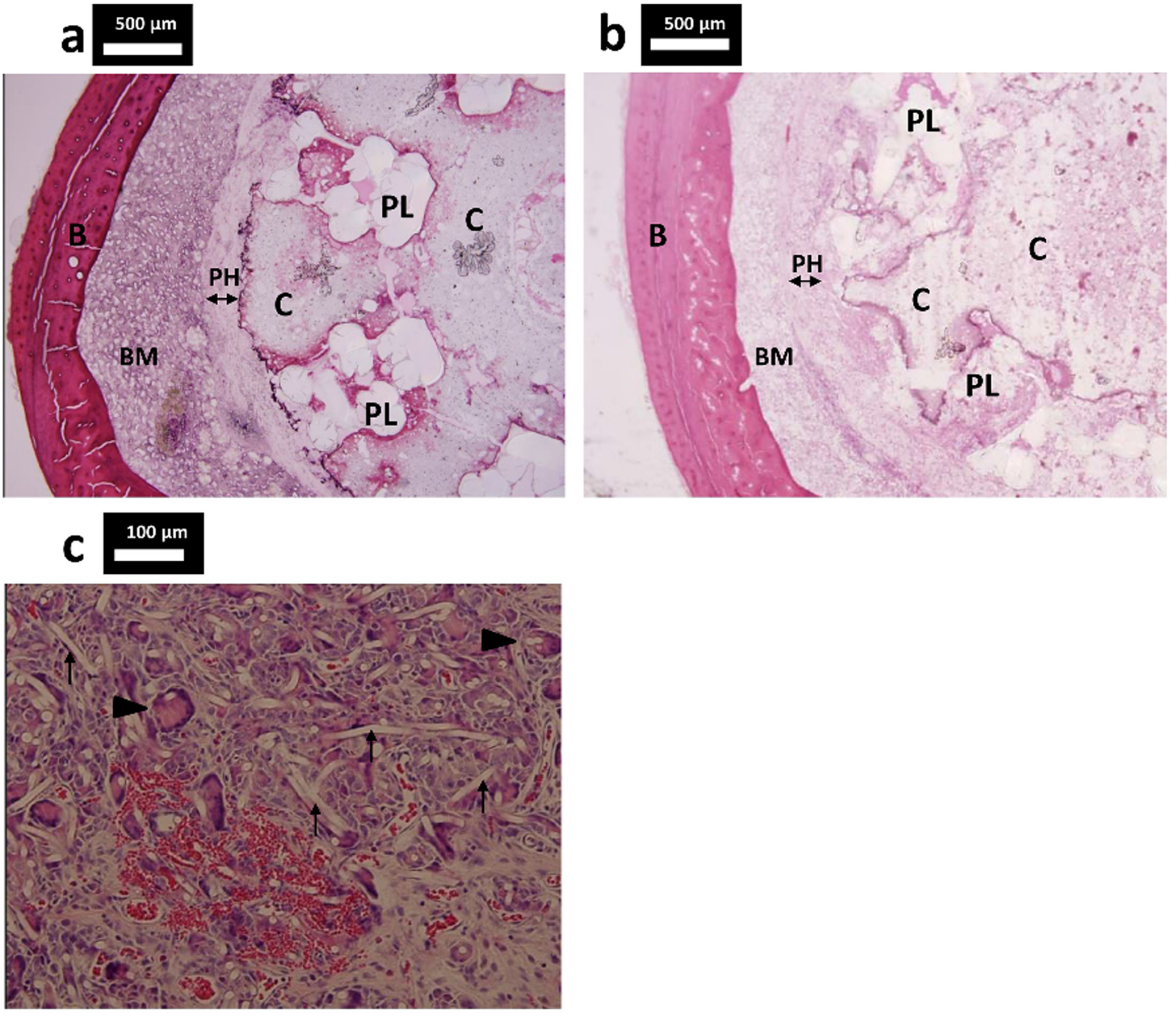
Histologic cross sections of specimens from the IM-BM group. Histologic cross sections of specimens from the IM-BM group (PLLA + CPC + PHA) stained with hematoxylin and eosin. (a) A PHA fiber mat layer surrounded the CPC, and the PLLA tube was not degraded at week 20. (b) At week 52, the PLLA tube seemed to have degraded gradually. (c) Multinucleated giant cell (arrowhead) and neovascularization were observed in the PHA fiber mat layer at week 52. PHA fiber (arrow). PL: PLLA woven tube; PH: PHA fiber mat C: CPC; B: bone cortex; BM: bone marrow.

Histologic examination at week 20 in the IM-BM group indicated that the PLLA tube was not degraded yet (Figure 8a). Histologic examination at week 52 in the IM-BM group showed that the PLLA tube seemed to have degraded gradually (Figure 8b). A multinucleated giant cell and neovascularization were observed in the PHA fiber mat layer (Figure 8c).

## Discussion

The incidence of fragility fractures has been increasing in developed countries. Since Galibert et al. in 1987 introduced percutaneous poly (methyl methacrylate) (PMMA)-assisted vertebroplasty [15], many authors have reported treatment outcomes for vertebroplasty or balloon kyphoplasty. PMMA has excellent compressive, bending, and tensile strength [16]. However, PMMA monomer toxicity produces a risk of hypotension [17], and can damage surrounding cells due to heat evolution during the hardening process. In addition to these problems, PMMA is not osteoconductive and does not enhance bone remodeling.

Alternatively, CPC (Biopex) is one of the most commonly used injectable bone cement pastes and is highly biocompatible, with excellent osteoconductivity [12]. The cement is absorbed progressively from the outer surface and replaced by bone tissue through the normal remodeling process [12]. Moreover, it can set without heat evolution. However, CPC is brittle and has generally low mechanical strength [3], [4], except for compressive strength. Thus far, CPC has been mainly limited to vertebroplasty and balloon kyphoplasty, which require a high compressive strength.

We inserted a PLLA woven tube into the intramedullary cavity with injection of CPC paste in order to overcome the above-mentioned problems. We wrapped the PLLA tube with nonwoven PHA fiber mat to prevent both CPC leakage from the fracture site and embolism. The PHA used in the present work was comprised of the copolymers 3HB and 18% 4HB. We also attempted to use a nonwoven poly(lactic-co-glycolic acid) (PLGA) fiber mat as a leakage prevention material in a preliminary trial with acrylic tubes. However, the PLGA mat did not extend far enough into the cavity and easily ruptured during injection of CPC paste. In contrast, the present animal experiment demonstrated that a nonwoven PHA fiber mat wrapped around a PLLA tube can efficiently prevent cement leakage from the fracture site and allow almost complete filling of the reamed cavity with CPC, as observed by postoperative soft X-ray analysis as well as histologic examination (Figures 7a and 8a).

This animal experiment also demonstrated that IM-BM markedly improved mechanical strength when compared with CPC only. Rabbits in the IM-BM group had no fracture displacement, whereas three of four rabbits in the CPC group had fracture displacements within 1 week after the surgery. Furthermore, the IM-BM also revealed the better results than other two groups (PLLA + CPC, K-wire). Two of four rabbits in the PLLA + CPC group and three of four rabbits in the K-wire group suffered fracture displacement, and one of four rabbits in the K-wire group suffered cutout at postoperative week 1. Histologic examination at week 52 in the IM-BM group showed partial degradation of the PLLA tube in the CPC. It has been previously reported that it takes 1.5–5 years for the degradation of PLLA to be complete, as shown in Table 3 [7]. The absorption of the nonwoven PHA fiber mat is preferable, because it enables the CPC to make contact with the bone gradually. However, partial absorption of the nonwoven PHA fiber mat was unclear at weeks 20 and 52. A multinucleated giant cell and neovascularization were observed in the PHA fiber mat layer. This suggests that the PHA used in our study have good biocompatibility. We are now conducting a long-term study to evaluate the degradation behavior of PHA.

**Table 3:** Properties of biodegradable thermoplastic polyesters. (Wu Q. 2009 [7]).

	Tm (°C)	Tg (°C)	Tensile strength (MPa)	Tensile modulus(GPa)	Elongation atbreak (%)	Absorption rate
PGA	225	35	70	6900	<3	6 weeks
PLLA	175	65	28–50	1200–2700	6	1.5–5 years
PDLLA	Amorphous	50–55	29–35	1900–2400	6	3 months
P(3HB)	180	1	36	2500	3	2 years
P(3HB-co-4HB)(4HB 16%)	152	−8	26	Not measured	444	Unknown

PGA: poly glycolic acid; PLLA: poly(l-lactide); PDLLA: poly(_D L_–lactide); P(3HB): poly(3-hydroxybutyrate); P(3HB-co-4HB) (4HB 16%): poly(3-hydroxybutyrate-co-16% 4-hydroxybutyrate); Tm: melting temperature; Tg: glass transition temperature.

Mechanical testing has clearly shown that the combination of the PLLA woven tube with CPC and PHA fiber mat improved the apparent mechanical properties, including a significant increase in fracture energy and flexural strength. All the stress–strain curves for group 1 (PLLA + CPC + PHA) showed slope changes at the end of zone 1 (Figure 6). The change may be due to fracture of bonding at the interface between the CPC and the PHA fiber mat. Thus, cracks in the CPC followed the fracture of bonding. The slope in zone 2 was smaller than that in zone 1. It is thought that the PLLA woven tube mainly supported the load with the CPC, because the PHA fiber mat prevented the CPC from breaking into pieces. In zone 3, the slope became flat. The PLLA woven tube was believed to be considerably deformed. In zone 4, the slope became negative. The CPC was not able to support the load any further because it was broken into pieces.

It should be emphasized that the average fracture energy for the IM-BM group (PLLA + CPC + PHA) was 60 to 200 times greater than that of CPC + PHA group and CPC group in our mechanical testing. Considering the result that the mean fracture energy of PLLA + CPC + PHA group (1210±334 J/m^2^) was 3 times higher (*p*<0.001) than that of the PLLA + CPC group (434±63 J/m^2^), the enhanced fracture energy of PLLA + PHA + CPC implant was mainly due to the PLLA tube, but PHA fiber mat also improved the fracture energy significantly.

The maximum flexural strength of K-wire fixation in our previous mechanical study using two 1.6-mm K-wires was almost same as that of PLLA + PHA + CPC group in our current study (3.0 MPa in K-wire group vs 2.71 MPa in PLLA + PHA + CPC group). However, we think that IM-BM was strong enough to reinforce and stabilize incomplete fractures even in the distal thigh where bone is exposed to the highest bending and torsional stresses. In fact, a benefit of the IM-BM implant is that it is not harder than necessary and the risk of cutout is low, whereas metallic implants sometimes cause cutout in severely osteoporotic patients. The current study indicates that IM-BM can also be applied to insufficiency fractures in other areas in the body such as rib and proximal humerus.

## Conclusions

In conclusion, the present work demonstrated that IM-BM was strong enough to reinforce and stabilize incomplete fractures with both mechanical testing and an animal experiment even in the distal thigh, where bone is exposed to the highest bending and torsional stresses in the body. The combination of three biomaterials with different physical and biological properties can be one treatment option for those with severe osteoporosis.
